# Supplementary material for: Causal Relationship between Aging and Anorexia Nervosa: A White-Matter-Microstructure-Mediated Mendelian Randomization Analysis
Source: Biomedicines. 2024 Aug 16;12(8):1874. doi: 10.3390/biomedicines12081874 (PMC11351342; doi:10.3390/biomedicines12081874)
Supplement: Supplementary file 1 [file biomedicines-12-01874-s001.zip › Supplementary Figure/Figure S4/Singe SNP plot of MR analysis for TL to ubm-b-1882.pdf]

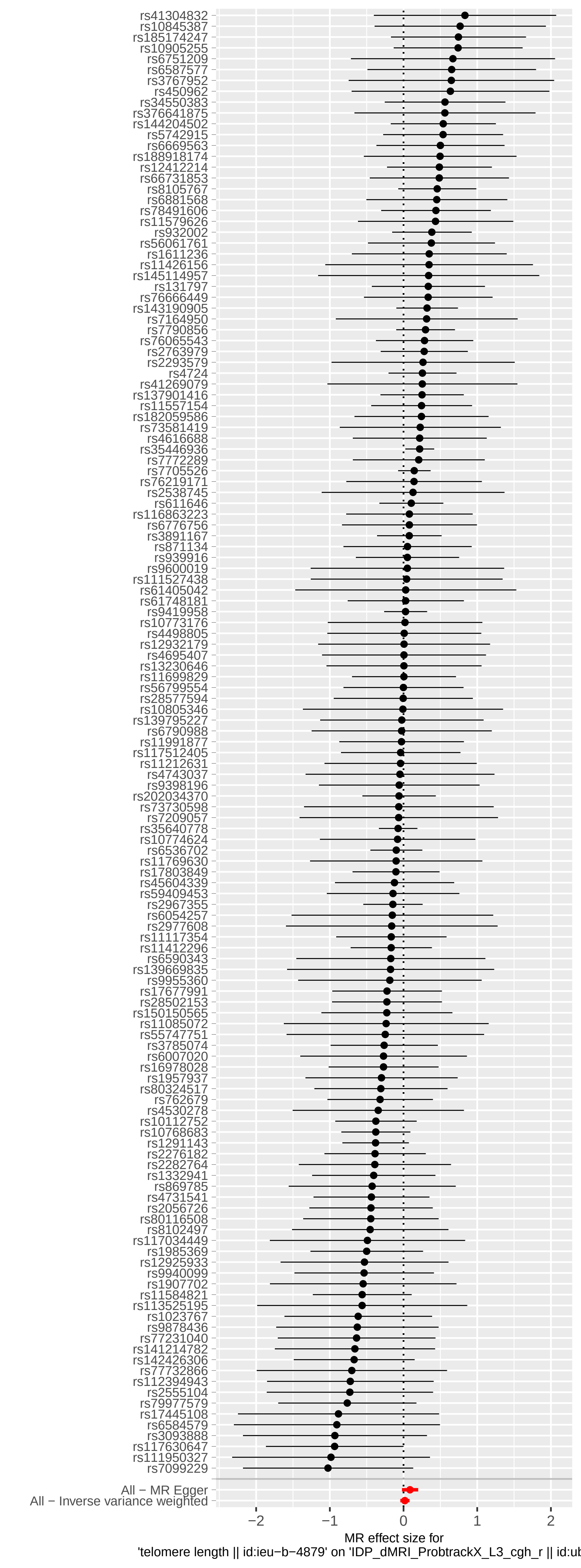
rs41304832
rs10845387
rs185174247
rs10905255
rs6751209
rs6587577
rs3767952
rs450962
rs34550383
rs376641875
rs144204502
rs5742915
rs6669563
rs188918174
rs12412214
rs66731853
rs8105767
rs6881568
rs78491606
rs11579626
rs932002
rs56061761
rs1611236
rs11426156
rs145114957
rs131797
rs76666449
rs143190905
rs7164950
rs7790856
rs76065543
rs2763979
rs2293579
rs4724
rs41269079
rs137901416
rs11557154
rs182059586
rs73581419
rs4616688
rs35446936
rs7772289
rs7705526
rs76219171
rs2538745
rs611646
rs116863223
rs6776756
rs3891167
rs871134
rs939916
rs9600019
rs111527438
rs61405042
rs61748181
rs9419958
rs10773176
rs4498805
rs12932179
rs4695407
rs13230646
rs11699829
rs56799554
rs28577594
rs10805346
rs139795227
rs6790988
rs11991877
rs117512405
rs11212631
rs4743037
rs9398196
rs202034370
rs73730598
rs7209057
rs35640778
rs10774624
rs6536702
rs11769630
rs17803849
rs45604339
rs59409453
rs2967355
rs6054257
rs2977608
rs11117354
rs11412296
rs6590343
rs139669835
rs9955360
rs17677991
rs28502153
rs150150565
rs11085072
rs55747751
rs3785074
rs6007020
rs16978028
rs1957937
rs80324517
rs762679
rs4530278
rs10112752
rs10768683
rs1291143
rs2276182
rs2282764
rs1332941
rs869785
rs4731541
rs2056726
rs80116508
rs8102497
rs117034449
rs1985369
rs12925933
rs9940099
rs1907702
rs11584821
rs113525195
rs1023767
rs9878436
rs77231040
rs141214782
rs142426306
rs77732866
rs112394943
rs2555104
rs79977579
rs17445108
rs6584579
rs3093888
rs117630647
rs111950327
rs7099229
All − MR Egger
All − Inverse variance weighted
−2
−1
0
1
2
MR effect size for
'telomere length || id:ieu−b−4879' on 'IDP_dMRI_ProbtrackX_L3_cgh_r || id:ub
